# Supplementary figures and images for: Impact of the yeast S0/uS2-cluster ribosomal protein rpS21/eS21 on rRNA folding and the architecture of small ribosomal subunit precursors
Source: PLoS One. 2023 Mar 30;18(3):e0283698. doi: 10.1371/journal.pone.0283698 (PMC10062582; doi:10.1371/journal.pone.0283698)

**A**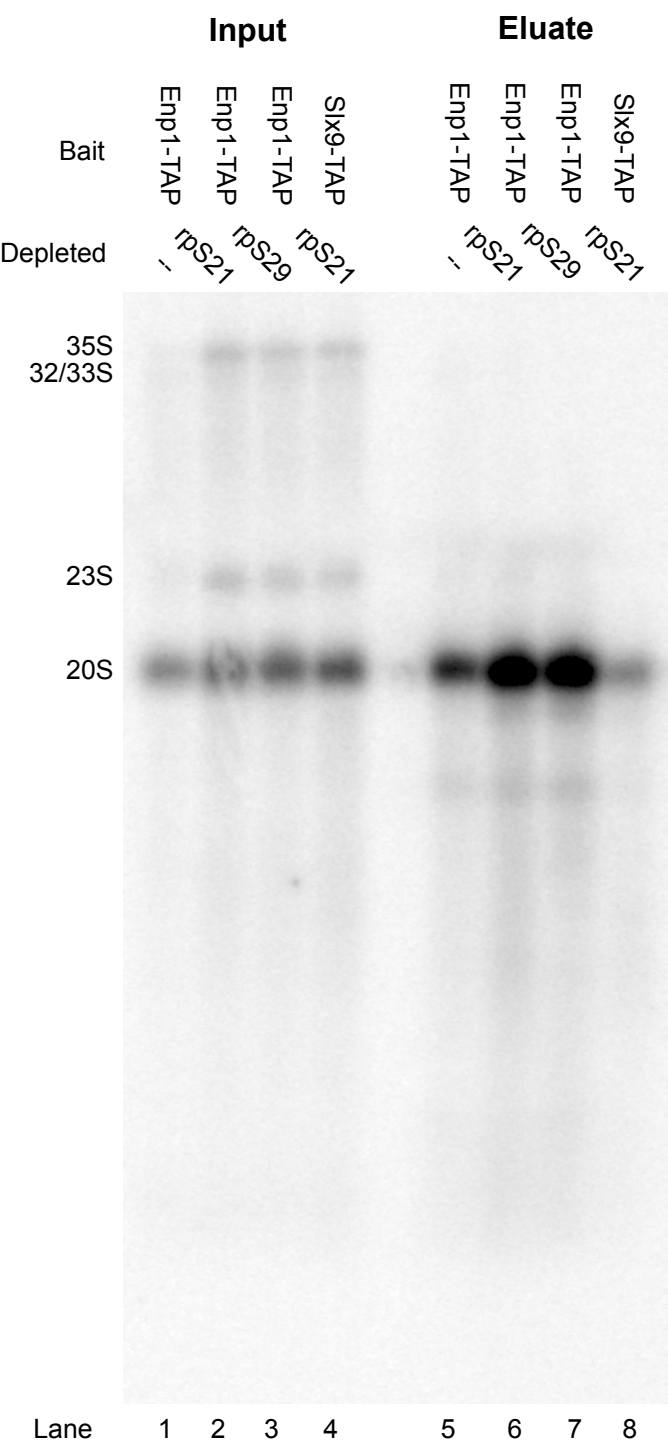**B**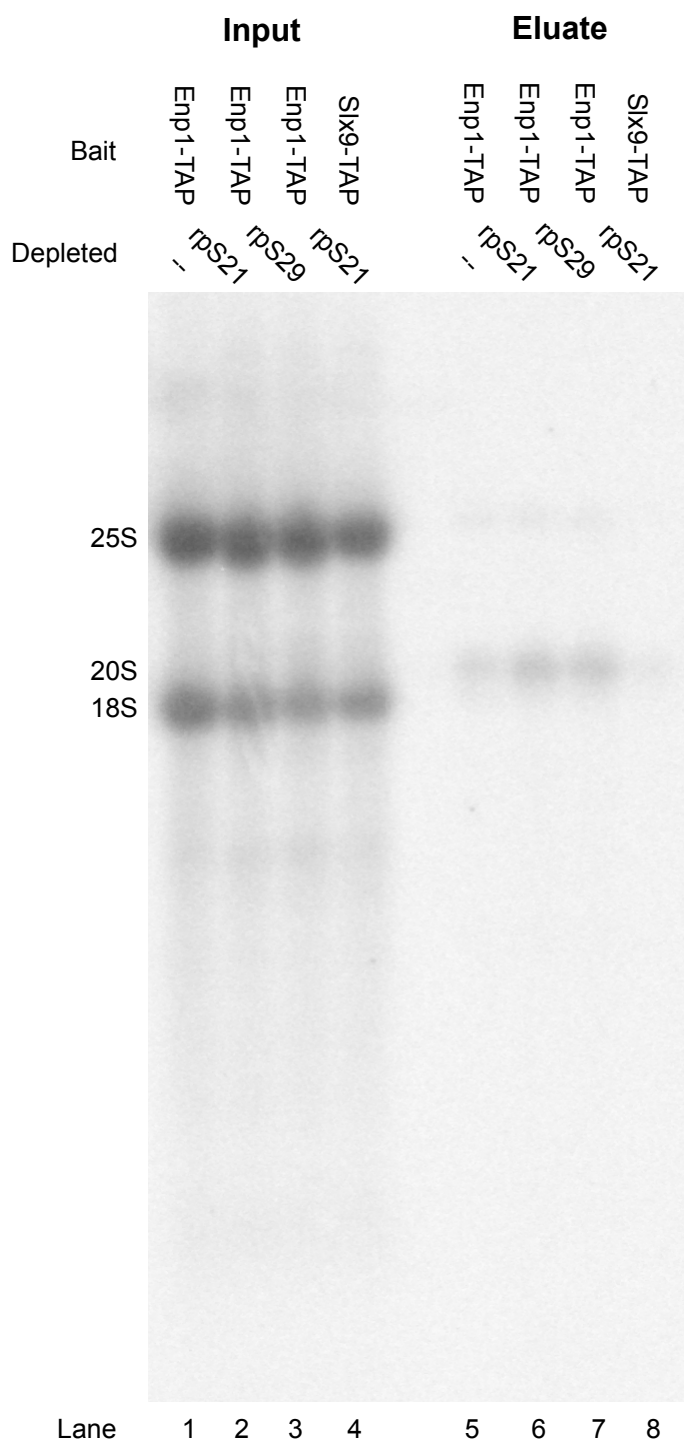**C**

| rRNA in | Normalized ratio<br>20S:18S | Normalized ratio<br>25S:18S |
|---------|-----------------------------|-----------------------------|
| Lane 1  | 1,00                        | 1,00                        |
| Lane 2  | 1,62                        | 1,31                        |
| Lane 3  | 2,20                        | 1,44                        |
| Lane 4  | 2,09                        | 1,24                        |

Supplement: S1 Appendix — Cells of yeast strains Y2226, Y2109, Y2110, and Y3153 which express the indicated r-protein (‘depleted’) under control of the GAL1/10 promoter, and the indicated biogenesis factor (‘Bait’) in fusion with the TAP-tag were incubated for four hours in a glucose-containing medium (see Materials and methods). Corresponding cellular extracts were used for affinity purification of the bait proteins and (pre-)rRNA composition of the extracts (‘Input’) and final eluates (‘Eluate’) were analyzed by RNA extraction and northern blotting with probes O1819 (A) and a mixture of probes O205 and O212 (B). On the left of (A) and (B), the detected (pre-)rRNA species are designated. The ratios indicated in (C) of the 20S pre-rRNA to 18S rRNA and of the 25S rRNA to 18S rRNA ratio in the input fractions were determined with ImageJ. The conditional expression mutant of the S3-cluster r-protein rpS29/uS14 (lanes 3 and 7) was included for comparison. (PDF) [file pone.0283698.s001.pdf]

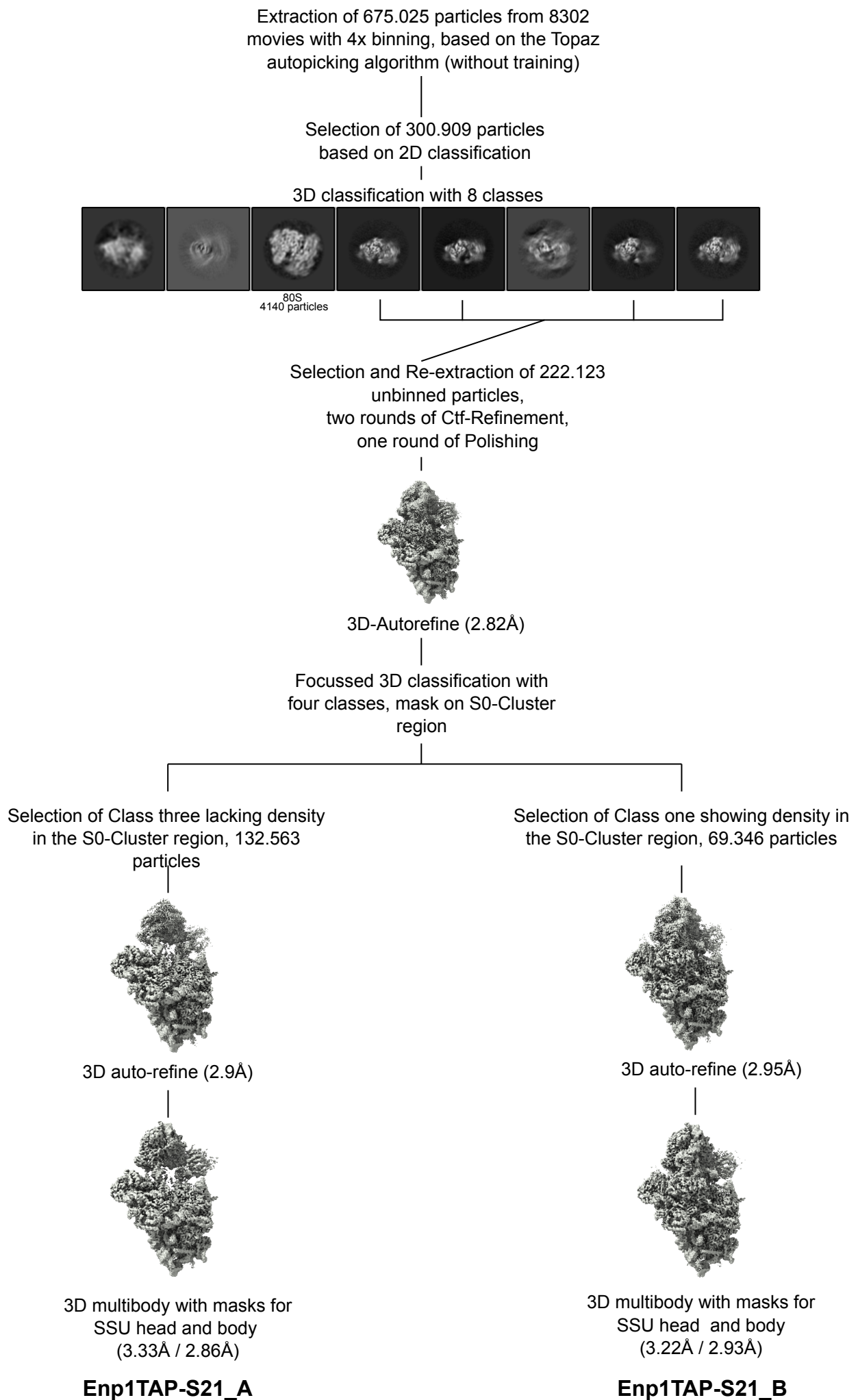

Supplement: S4 Appendix — (PDF) [file pone.0283698.s004.pdf]
