# Supplementary material for: Impact of the yeast S0/uS2-cluster ribosomal protein rpS21/eS21 on rRNA folding and the architecture of small ribosomal subunit precursors
Source: PLoS One. 2023 Mar 30;18(3):e0283698. doi: 10.1371/journal.pone.0283698 (PMC10062582; doi:10.1371/journal.pone.0283698)
Supplement: S2 Appendix — (PDF) [file pone.0283698.s002.pdf]

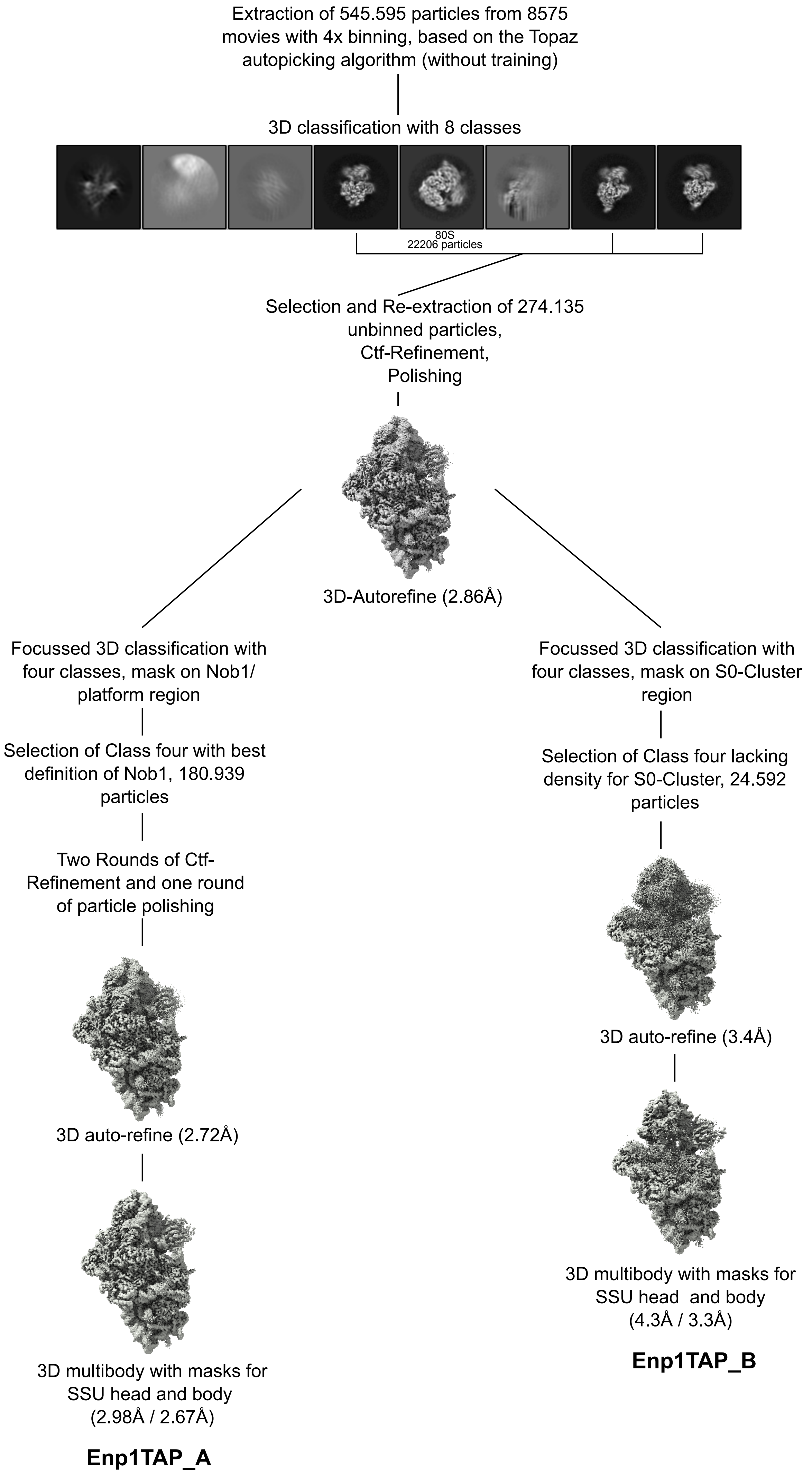

Extraction of 545.595 particles from 8575 movies with 4x binning, based on the Topaz autopicking algorithm (without training)
3D classification with 8 classes
80S
22206 particles
Selection and Re-extraction of 274.135 unbinned particles, Ctf-Refinement, Polishing
3D-Autorefine (2.86Å)
Focussed 3D classification with four classes, mask on Nob1/ platform region
Selection of Class four with best definition of Nob1, 180.939 particles
Two Rounds of Ctf-Refinement and one round of particle polishing
3D auto-refine (2.72Å)
3D multibody with masks for SSU head and body (2.98Å / 2.67Å)
Enp1TAP_A
Focussed 3D classification with four classes, mask on S0-Cluster region
Selection of Class four lacking density for S0-Cluster, 24.592 particles
3D auto-refine (3.4Å)
3D multibody with masks for SSU head and body (4.3Å / 3.3Å)
Enp1TAP_B
