# Supplementary material for: Impact of the yeast S0/uS2-cluster ribosomal protein rpS21/eS21 on rRNA folding and the architecture of small ribosomal subunit precursors
Source: PLoS One. 2023 Mar 30;18(3):e0283698. doi: 10.1371/journal.pone.0283698 (PMC10062582; doi:10.1371/journal.pone.0283698)
Supplement: S5 Appendix — (PDF) [file pone.0283698.s005.pdf]

name	chain	modeled_residues Enp1TAP_A	mapped_contacts Enp1TAP_A	modeled_residues Enp1TAP-S21_A	mapped_contacts Enp1TAP-S21_A
rpS0	P	206 of 252 res.:<br>2-207;	rpS2(8);<br>rpS17(16);<br>RpS21(20);<br><br>Nob1(18);<br>H26(2);<br>H35(3);<br>H36(2);<br>H37(1);	-	-
rpS1	Q	214 of 255 res.:<br>20-233;	rpS14(5);<br><br>Pno1(2);<br>Nob1(2);<br>H22(5);<br>H23(5);<br>H23a(4);<br>H26(4);<br>H26_ES7(3);	214 of 255 res.:<br>20-233;	rpS14(5);<br><br>H22(4);<br>H23(4);<br>H23a(4);<br>H26(3);<br>H26_ES7(3);
rpS2	R	207 of 254 res.:<br>35-87;<br>96-249;	rpS0(6);<br>rpS9(5);<br>rpS21(16);<br>rpS22(6);<br><br>H1(6);<br>H2(1);<br>H25(3);<br>H26a(2);<br>H35(1);<br>H36(2);	-	-
rpS4	S	260 of 261 res.:<br>2-261;	rpS6(6);<br>rpS9(4);<br>rpS24(8);<br><br>H6a(4);<br>H7(8);<br>H9(4);<br>H9_ES3b(1);<br>H12(3);<br>H13(4);<br>H15(5);<br>H21(8);<br>H21_unk(2);<br>H21_ES6a(2);	260 of 261 res.:<br>2-261;	rpS6(5);<br>rpS9(5);<br>rpS24(7);<br><br>H6a(4);<br>H7(10);<br>H9(5);<br>H9_ES3b(1);<br>H12(2);<br>H13(3);<br>H15(5);<br>H21(9);<br>H21_unk(2);<br>H21_ES6a(2);
rpS5	B	201 of 225 res.:<br>20-149;<br>155-225;	rpS16(16);<br>rpS25(9);<br>rpS28(14);<br><br>H29(1);<br>H41(7);<br>H42(1);<br>H43(10);	201 of 225 res.:<br>20-149;<br>155-225;	rpS16(15);<br>rpS25(9);<br>rpS28(13);<br><br>H29(2);<br>H41(7);<br>H42(2);<br>H43(10);

rpS6	T	226 of 236 res.:<br>1-226;	rpS4(4);<br>rpS24(1);<br><br>H6(9);<br>H7(2);<br>H8(22);<br>H9_ES3a(3);<br>H9(1);<br>H9_ES3b(2);<br>H10(6);<br>H13(5);<br>H14(3);<br>H44(4);	226 of 236 res.:<br>1-226;	rpS4(4);<br><br>H6(10);<br>H7(2);<br>H8(22);<br>H9_ES3a(3);<br>H9(1);<br>H9_ES3b(2);<br>H10(6);<br>H13(5);<br>H14(3);<br>H44(3);
rpS7	U	184 of 190 res.:<br>4-187;	rpS13(2);<br>rpS22(8);<br><br>H20(2);<br>H21_ES6c(2);<br>H21_ES6b(2);<br>H21(4);<br>H21_ES6d(4);<br>H22(1);	184 of 190 res.:<br>4-187;	rpS13(3);<br>rpS22(8);<br><br>H20(2);<br>H21_ES6c(3);<br>H21_ES6b(2);<br>H21(5);<br>H21_ES6d(5);<br>H22(1);
rpS8	V	185 of 200 res.:<br>2-122;<br>135-198;	rpS11(8);<br><br>H6a(2);<br>H7(4);<br>H9_ES3a(3);<br>H9(9);<br>H11(14);<br>H13(8);<br>H44(6);	185 of 200 res.:<br>2-122;<br>135-198;	rpS11(10);<br><br>H6a(2);<br>H7(4);<br>H9_ES3a(2);<br>H9(9);<br>H11(15);<br>H13(8);<br>H44(6);
rpS9	W	185 of 197 res.:<br>2-186;	rpS2(5);<br>rpS4(4);<br>rpS24(2);<br>rpS30(5);<br><br>H1(2);<br>H3(1);<br>H4(8);<br>H12(2);<br>H16(2);<br>H17(8);<br>H18(4);<br>H21(4);<br>H21_unk(6);	185 of 197 res.:<br>2-186;	rpS4(4);<br>rpS24(2);<br>rpS30(5);<br><br>H1(1);<br>H3(2);<br>H4(7);<br>H12(1);<br>H16(1);<br>H17(8);<br>H18(4);<br>H21(4);<br>H21_unk(6);
rpS11	X	141 of 156 res.:<br>5-145;	rpS8(9);<br>rpS22(1);<br>rpS23(8);<br><br>H7(6);<br>H9(6);<br>H11(14);<br>H12(1);<br>H19(2);<br>H20(1);	141 of 156 res.:<br>5-145;	rpS8(15);<br>rpS22(2);<br>rpS23(8);<br><br>H7(5);<br>H9(5);<br>H11(14);<br>H12(1);<br>H19(2);<br>H20(1);<br>H21(1);

rpS13	Y	150 of 151 res.:<br>2-151;	rpS7(1);<br>rpS22(2);<br>rpS27(9);<br><br>H20(7);<br>H21_ES6d(2);<br>H22(21);<br>H23a(2);<br>H24(1);<br>H25(2);<br>H26(1);	150 of 151 res.:<br>2-151;	rpS7(2);<br>rpS22(2);<br>rpS27(9);<br><br>H20(7);<br>H21_ES6d(2);<br>H22(21);<br>H23a(2);<br>H24(1);<br>H25(2);
rpS14	Z	127 of 137 res.:<br>11-137;	rpS1(8);<br>Pno1(16);<br><br>H23(17);<br>H23a(1);<br>H24(3);<br>H45(4);	127 of 137 res.:<br>11-137;	rpS1(8);<br>Pno1(19);<br><br>H23(18);<br>H23a(1);<br>H24(3);<br>H45(4);
rpS15	E	114 of 142 res.:<br>14-69;<br>72-129;	rpS18(9);<br><br>H30(2);<br>H31_unk1(3);<br>H32(4);<br>H33(2);<br>H42(10);	114 of 142 res.:<br>14-69;<br>72-129;	rpS18(11);<br><br>H30(2);<br>H31_unk1(4);<br>H32(5);<br>H33(1);<br>H42(10);
rpS16	F	125 of 143 res.:<br>3-127;	rpS5(19);<br>rpS19(1);<br><br>H38(2);<br>H39(2);<br>H39_ES9(2);<br>H41(5);<br>H43(6);	125 of 143 res.:<br>3-127;	rpS5(20);<br><br>H39(1);<br>H39_ES9(2);<br>H41(5);<br>H43(4);
rpS17	C	61 of 136 res.:<br>65-125;	rpS0(18);<br>Nob1(1);	-	-
rpS18	H	122 of 146 res.:<br>10-131;	rpS15(11);<br>rpS19(5);<br>rpS25(2);<br><br>H41(3);<br>H42(10);	122 of 146 res.:<br>10-131;	rpS15(12);<br>rpS19(4);<br>rpS25(1);<br><br>H41(3);<br>H42(10);
rpS19	I	141 of 144 res.:<br>3-143;	rpS16(1);<br>rpS18(6);<br><br>H30(3);<br>H39_ES9(7);<br>H41(18);<br>H42(3);<br>H43(2);	141 of 144 res.:<br>3-143;	rpS18(5);<br><br>H30(3);<br>H39_ES9(7);<br>H41(20);<br>H42(2);<br>H43(2);
rpS21	a	86 of 87 res.:<br>1-86;	rpS0(16);<br>rpS2(17);<br>rpS22(2);<br>rpS27(5);<br><br>H26a(1);	-	-

rpS22	b	129 of 130 res.:<br>2-130;	rpS2(8);<br>rpS7(7);<br>rpS11(1);<br>rpS13(3);<br>rpS21(2);<br>rpS23(2);<br>rpS27(8);<br><br>H20(5);<br>H21_ES6c(2);<br>H21(8);<br>H22(3);<br>H25(11);<br>H26(1);	129 of 130 res.:<br>2-130;	rpS7(6);<br>rpS11(2);<br>rpS13(3);<br>rpS23(1);<br>rpS27(6);<br><br>H20(5);<br>H21_ES6c(3);<br>H21(9);<br>H22(3);<br>H25(11);<br>H26(1);
rpS23	c	144 of 145 res.:<br>2-145;	rpS11(6);<br>rpS22(1);<br>Tsr1(11);<br><br>H3(6);<br>H4(1);<br>H5(1);<br>H11(4);<br>H12(1);<br>H15(1);<br>H18(9);<br>H19(5);<br>H20(1);<br>H25(6);<br>H27(2);<br>H44(1);	144 of 145 res.:<br>2-145;	rpS11(6);<br>rpS22(1);<br>Tsr1(11);<br><br>H3(6);<br>H4(1);<br>H5(1);<br>H11(4);<br>H12(1);<br>H15(1);<br>H18(9);<br>H19(5);<br>H20(1);<br>H25(6);<br>H27(2);<br>H44(1);
rpS24	d	130 of 135 res.:<br>3-132;	rpS4(7);<br>rpS6(1);<br>rpS9(1);<br><br>H5(2);<br>H6(3);<br>H8(8);<br>H15(4);<br>H17(10);<br>H21_unk(1);<br>H21_ES6a(5);	130 of 135 res.:<br>3-132;	rpS4(7);<br>rpS9(1);<br><br>H5(2);<br>H6(3);<br>H8(9);<br>H15(5);<br>H17(10);<br>H21_unk(1);<br>H21_ES6a(4);
rpS25	K	69 of 108 res.:<br>36-104;	rpS5(8);<br>rpS18(2);<br><br>H41(6);	69 of 108 res.:<br>36-104;	rpS5(8);<br>rpS18(1);<br><br>H41(7);
rpS27	f	81 of 82 res.:<br>2-82;	rpS13(7);<br>rpS21(4);<br>rpS22(8);<br><br>H22(7);<br>H26(6);	81 of 82 res.:<br>2-82;	rpS13(7);<br>rpS22(9);<br><br>H22(7);<br>H26(6);
rpS28	L	60 of 67 res.:<br>6-65;	rpS5(15);<br><br>H28(2);<br>H43(1);	60 of 67 res.:<br>6-65;	rpS5(17);<br><br>H28(3);<br>H43(1);

rpS30	g	36 of 63 res.:<br>25-60;	rpS9(6);<br><br>H4(2);<br>H16(1);<br>H17(2);<br>H18(9);	30 of 63 res.:<br>25-47;<br>54-60;	rpS9(6);<br><br>0(14);<br>H4(2);<br>H16(2);<br>H18(10);
Enp1	e	200 of 483 res.:<br>213-267;<br>273-326;<br>331-361;<br>367-379;<br>383-394;<br>414-427;<br>435-446;<br>454-462;	 H32(2);<br>H34(1);	179 of 483 res.:<br>213-267;<br>273-326;<br>331-361;<br>367-379;<br>383-394;<br>414-427;	H32(1);<br>H33(1);
Pno1	p	180 of 274 res.:<br>93-272;	rpS1(2);<br>rpS14(12);<br><br>Nob1(8);<br>H24(2);<br>H45(8);	180 of 274 res.:<br>93-272;	rpS1(2);<br>rpS14(15);<br><br>H24(1);<br>H28(3);<br>H45(9);
Rio2	r	239 of 425 res.:<br>13-26;<br>31-42;<br>45-58;<br>77-92;<br>95-110;<br>120-126;<br>152-237;<br>247-287;<br>298-330;	Tsr1(1);<br><br>H29(3);<br>H30(3);<br>H42(2);	190 of 425 res.:<br>78-92;<br>95-110;<br>120-126;<br>152-156;<br>158-237;<br>247-287;<br>298-313;<br>321-330;	H29(3);<br>H30(4);<br>H42(2);
Tsr1	t	598 of 788 res.:<br>44-114;<br>119-268;<br>273-309;<br>335-350;<br>463-786;	rpS23(7);<br>Rio2(1);<br><br>H5(5);<br>H7(1);<br>H11(3);<br>H15(1);<br>H17(2);<br>H27(2);<br>H31_unk1(3);<br>H31(2);<br>H32(2);<br>H34(1);<br>H44(6);	619 of 788 res.:<br>12-32;<br>44-114;<br>119-268;<br>273-309;<br>335-350;<br>463-786;	rpS23(7);<br><br>H2(3);<br>H5(4);<br>H7(1);<br>H11(3);<br>H15(1);<br>H17(1);<br>H19(3);<br>H24(1);<br>H27(5);<br>H31_unk1(3);<br>H32(2);<br>H44(4);<br>H45(7);
Nob1	o	314 of 459 res.:<br>7-118;<br>221-249;<br>261-419;<br>444-457;	rpS0(18);<br>rpS1(3);<br>rpS17(1);<br>Pno1(11);<br><br>H23a(1);<br>H26(9);<br>H26a(2);<br>H35(1);<br>H36(4);<br>H37(4);<br>H45(1);	-	-
