# Supplementary material for: Impact of the yeast S0/uS2-cluster ribosomal protein rpS21/eS21 on rRNA folding and the architecture of small ribosomal subunit precursors
Source: PLoS One. 2023 Mar 30;18(3):e0283698. doi: 10.1371/journal.pone.0283698 (PMC10062582; doi:10.1371/journal.pone.0283698)
Supplement: S6 Appendix — (PDF) [file pone.0283698.s006.pdf]

Extraction of 318.619 particles from 6462
movies with 4x binning, based on the Topaz
autopicking algorithm (without training)

3D classification with 8 classes

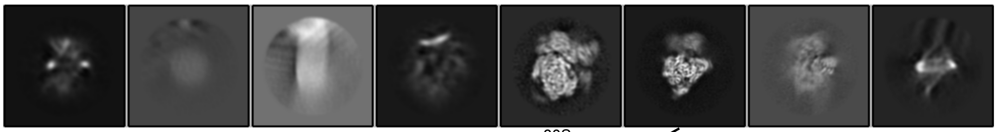

80S
10573 particles

Selection and Re-extraction of 53.304
unbinned particles,
one round of Ctf-Refinement,
one round of Polishing

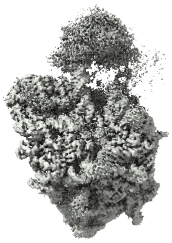

3D-Autorefine (3.3Å)

Slx9TAP-S21_A
