# Supplementary material for: Impact of the yeast S0/uS2-cluster ribosomal protein rpS21/eS21 on rRNA folding and the architecture of small ribosomal subunit precursors
Source: PLoS One. 2023 Mar 30;18(3):e0283698. doi: 10.1371/journal.pone.0283698 (PMC10062582; doi:10.1371/journal.pone.0283698)
Supplement: S7 Appendix — (PDF) [file pone.0283698.s007.pdf]

Helix	modeled_residues Enp1TAP_A	mapped_contacts Enp1TAP_A	modeled_residues Enp1TAP-S21_A	mapped_contacts Enp1TAP-S21_A
H1	13 of 14<br>2-8;<br>15-20;	rpS2(7);<br>rpS9(2);<br><br>H2(4);<br>H3(2);<br>H12(1);<br>H18(4);<br>H19(2);<br>H27(1);	13 of 14<br>2-8;<br>15-20;	rpS9(1);<br><br>H2(4);<br>H3(2);<br>H12(1);<br>H18(3);<br>H19(2);<br>H27(1);
H2	14 of 14<br>9-14;<br>1137-1144;	rpS2(3);<br><br>H1(5);<br>H19(2);<br>H26a(3);<br>H27(1);<br>H28(1);<br>H36(4);	14 of 14<br>9-14;<br>1137-1144;	Tsr1(2);<br><br>H1(5);<br>H19(2);<br>H26a(3);<br>H27(1);<br>H28(2);
H3	23 of 23<br>21-31;<br>595-606;	rpS9(1);<br>rpS23(8);<br><br>H1(1);<br>H4(2);<br>H12(4);<br>H15(1);<br>H18(3);<br>H19(1);	23 of 23<br>21-31;<br>595-606;	rpS9(2);<br>rpS23(8);<br><br>H1(2);<br>H4(1);<br>H12(3);<br>H15(1);<br>H18(3);
H4	23 of 23<br>32-41;<br>466-478;	rpS9(14);<br>rpS23(1);<br>rpS30(6);<br><br>H3(1);<br>H15(3);<br>H17(6);<br>H18(2);<br>H21_unk(2);	23 of 23<br>32-41;<br>466-478;	rpS9(10);<br>rpS23(1);<br>rpS30(6);<br><br>H3(1);<br>H15(3);<br>H17(5);<br>H18(3);<br>H21_unk(2);
H5	25 of 25<br>42-55;<br>424-434;	rpS23(1);<br>rpS24(3);<br>Tsr1(4);<br><br>H6a(5);<br>H7(1);<br>H12(1);<br>H13(2);<br>H15(6);	25 of 25<br>42-55;<br>424-434;	rpS23(1);<br>rpS24(3);<br>Tsr1(3);<br><br>H6a(5);<br>H7(1);<br>H12(1);<br>H13(2);<br>H15(5);
H6	34 of 37<br>56-72;<br>76-92;	rpS6(17);<br>rpS24(3);<br><br>H8(5);<br>H10(4);<br>H13(3);<br>H15(6);	34 of 37<br>56-72;<br>76-92;	rpS6(18);<br>rpS24(4);<br><br>H8(5);<br>H10(4);<br>H13(3);<br>H15(5);

H6a	11 of 11<br>93-99;<br>383-386;	rpS4(7);<br>rpS8(3);<br><br>H5(4);<br>H7(2);<br>H12(3);<br>H13(3);<br>H15(1);	11 of 11<br>93-99;<br>383-386;	rpS4(7);<br>rpS8(3);<br><br>H5(4);<br>H7(2);<br>H12(3);<br>H13(3);<br>H15(1);
H7	50 of 58<br>100-129;<br>289-308;	rpS4(8);<br>rpS6(5);<br>rpS8(4);<br>rpS11(12);<br>Tsr1(1);<br><br>H5(1);<br>H6a(2);<br>H8(1);<br>H9_ES3a(2);<br>H9(6);<br>H11(6);<br>H12(3);<br>H13(1);<br>H21(2);	50 of 58<br>100-129;<br>289-308;	rpS4(10);<br>rpS6(4);<br>rpS8(5);<br>rpS11(9);<br>Tsr1(1);<br><br>H5(1);<br>H6a(2);<br>H8(1);<br>H9_ES3a(2);<br>H9(6);<br>H11(6);<br>H12(2);<br>H13(1);<br>H21(2);
H8	38 of 38<br>138-175;	rpS6(31);<br>rpS24(6);<br><br>H6(5);<br>H7(1);<br>H9_ES3a(1);<br>H10(2);<br>H14(5);<br>H44(1);	38 of 38<br>138-175;	rpS6(28);<br>rpS24(7);<br><br>H6(5);<br>H7(1);<br>H9_ES3a(1);<br>H10(2);<br>H14(6);
H9_ES3a	19 of 28<br>176-187;<br>197-203;	rpS6(3);<br>rpS8(3);<br><br>H7(2);<br>H8(1);<br>H10(1);	19 of 28<br>176-187;<br>197-203;	rpS6(3);<br>rpS8(2);<br><br>H7(2);<br>H8(1);<br>H10(1);
H9	38 of 38<br>204-221;<br>246-265;	rpS4(7);<br>rpS6(1);<br>rpS8(11);<br>rpS11(11);<br><br>H7(7);<br>H9_ES3b(3);<br>H10(1);<br>H21_ES6d(10);	38 of 38<br>204-221;<br>246-265;	rpS4(8);<br>rpS6(1);<br>rpS8(11);<br>rpS11(11);<br><br>H7(7);<br>H9_ES3b(3);<br>H10(1);<br>H21_ES6d(10);
H9_ES3b	4 of 23<br>241-244;	rpS4(1);<br>rpS6(2);<br><br>H9(4);	5 of 23<br>222-222;<br>241-244;	rpS4(1);<br>rpS6(3);<br><br>H9(4);<br>H21_ES6d(3);

H10	19 of 23<br>266-276;<br>281-288;	rpS6(5);<br><br>H6(2);<br>H8(6);<br>H9_ES3a(1)<br>H9(1);	19 of 23<br>266-276;<br>281-288;	rpS6(5);<br><br>H6(2);<br>H8(6);<br>H9_ES3a(1);<br>H9(1);
H11	52 of 52<br>309-360;	rpS8(23);<br>rpS11(18);<br>rpS23(5);<br>Tsr1(3);<br><br>H7(6);<br>H12(1);<br>H20(1);<br>H27(3);	52 of 52<br>309-360;	rpS8(22);<br>rpS11(15);<br>rpS23(4);<br>Tsr1(3);<br><br>H7(6);<br>H12(1);<br>H20(1);<br>H27(2);
H12	23 of 23<br>361-383;	rpS4(2);<br>rpS9(3);<br>rpS11(1);<br>rpS23(1);<br><br>H1(1);<br>H3(5);<br>H5(1);<br>H6a(2);<br>H7(2);<br>H11(1);<br>H19(3);<br>H21(4);	23 of 23<br>361-383;	rpS4(2);<br>rpS9(3);<br>rpS11(1);<br>rpS23(1);<br><br>H1(1);<br>H3(3);<br>H5(1);<br>H6a(2);<br>H7(1);<br>H11(1);<br>H19(3);<br>H21(4);
H13	22 of 22<br>388-409;	rpS4(1);<br>rpS6(4);<br>rpS8(9);<br><br>H5(2);<br>H6(3);<br>H6a(1);<br>H7(1);<br>H14(2);<br>H44(7);	22 of 22<br>388-409;	rpS4(1);<br>rpS6(3);<br>rpS8(9);<br><br>H5(1);<br>H6(3);<br>H6a(1);<br>H7(1);<br>H14(2);<br>H44(7);
H14	14 of 14<br>410-423;	rpS6(2);<br><br>H8(3);<br>H13(1);	14 of 14<br>410-423;	rpS6(2);<br><br>H8(3);<br>H13(1);
H15	31 of 31<br>435-465;	rpS4(8);<br>rpS23(4);<br>rpS24(4);<br>Tsr1(1);<br><br>H3(1);<br>H4(2);<br>H5(13);<br>H6(9);<br>H6a(2);<br>H17(2);	31 of 31<br>435-465;	rpS4(8);<br>rpS23(4);<br>rpS24(4);<br>Tsr1(1);<br><br>H3(1);<br>H4(2);<br>H5(13);<br>H6(9);<br>H6a(2);<br>H17(3);
H16	17 of 31<br>479-487;<br>501-505;<br>507-509;	rpS9(2);<br>rpS30(1);<br><br>H17(1);	17 of 31<br>479-487;<br>501-505;<br>507-509;	rpS9(1);<br>rpS30(2);<br><br>H17(1);

H17	34 of 34<br>510-543;	rpS9(10);<br>rpS24(11);<br>rpS30(2);<br>Tsr1(2);<br><br>H4(4);<br>H15(5);<br>H16(1);<br>H18(1);	34 of 34<br>510-543;	rpS9(11);<br>rpS24(12);<br>Tsr1(1);<br><br>H4(3);<br>H15(6);<br>H16(1);<br>H18(1);
H18	51 of 51<br>544-594;	rpS9(4);<br>rpS23(10);<br>rpS30(7);<br><br>H1(4);<br>H3(3);<br>H4(6);<br>H17(2);	47 of 51<br>544-564;<br>567-577;<br>580-594;	rpS9(4);<br>rpS23(10);<br>rpS30(9);<br><br>H1(2);<br>H3(3);<br>H4(5);<br>H17(2);
H19	20 of 20<br>607-622;<br>1104-1107;	rpS11(3);<br>rpS23(9);<br><br>H1(2);<br>H2(4);<br>H3(1);<br>H12(2);<br>H20(2);<br>H25(6);<br>H26a(4);	20 of 20<br>607-622;<br>1104-1107;	rpS11(2);<br>rpS23(10);<br>Tsr1(3);<br><br>H1(3);<br>H2(4);<br>H12(2);<br>H20(3);<br>H25(6);<br>H26a(4);
H20	27 of 27<br>623-638;<br>966-976;	rpS7(3);<br>rpS11(1);<br>rpS13(6);<br>rpS22(6);<br>rpS23(3);<br><br>H11(1);<br>H22(5);<br>H23a(3);<br>H24(3);<br>H25(5);	27 of 27<br>623-638;<br>966-976;	rpS7(3);<br>rpS11(1);<br>rpS13(5);<br>rpS22(6);<br>rpS23(2);<br><br>H11(1);<br>H22(5);<br>H23a(3);<br>H24(2);<br>H25(6);
H21_ES6c	26 of 55<br>639-652;<br>682-693;	rpS7(7);<br>rpS22(2);<br><br>H21_ES6b(1);	26 of 55<br>639-652;<br>682-693;	rpS7(8);<br>rpS22(2);<br><br>H21_ES6b(1);
H21_ES6b	2 of 49<br>694-695;	rpS7(4);<br>1(2);<br>H21_ES6c(2);	2 of 49<br>694-695;	rpS7(4);<br>1(2);<br>H21_ES6c(2);
H21	44 of 44<br>743-764;<br>789-810;	rpS4(8);<br>rpS7(3);<br>rpS9(4);<br>rpS22(9);<br><br>H7(2);<br>H12(4);<br>H21_unk(2);<br>H21_ES6a(1);	44 of 44<br>743-764;<br>789-810;	rpS4(8);<br>rpS7(3);<br>rpS9(4);<br>rpS11(1);<br>rpS22(10);<br><br>H7(2);<br>H12(4);<br>H21_unk(2);<br>H21_ES6a(1);

H21_unk	9 of 9<br>765-773;	rpS4(3);<br>rpS9(14);<br>rpS24(1);<br><br>H4(2);<br>H21(2);<br>H21_ES6a(2);	9 of 9<br>765-773;	rpS4(3);<br>rpS9(11);<br>rpS24(1);<br><br>H4(2);<br>H21(2);<br>H21_ES6a(2);
H21_ES6a	12 of 15<br>774-779;<br>783-788;	rpS4(3);<br>rpS24(3);<br><br>H21(1);<br>H21_unk(2);	12 of 15<br>774-779;<br>783-788;	rpS4(4);<br>rpS24(3);<br><br>H21(1);<br>H21_unk(2);
H21_ES6d	44 of 49<br>811-833;<br>839-859;	rpS7(7);<br>rpS13(2);<br><br>H9(5);	44 of 49<br>811-833;<br>839-859;	rpS7(7);<br>rpS13(2);<br><br>H9(5);<br>H9_ES3b(1);
H22	45 of 45<br>860-883;<br>945-965;	rpS1(3);<br>rpS7(1);<br>rpS13(34);<br>rpS22(4);<br>rpS27(9);<br><br>H20(4);<br>H23(2);<br>H23a(6);<br>H26(3);	45 of 45<br>860-883;<br>945-965;	rpS1(3);<br>rpS7(1);<br>rpS13(32);<br>rpS22(4);<br>rpS27(10);<br><br>H20(4);<br>H23(2);<br>H23a(6);<br>H26(3);
H23	45 of 45<br>884-928;	rpS1(7);<br>rpS14(19);<br><br>H22(1);<br>H24(11);	45 of 45<br>884-928;	rpS1(4);<br>rpS14(20);<br><br>H22(1);<br>H24(11);
H23a	16 of 16<br>929-944;	rpS1(5);<br>rpS13(2);<br>rpS14(1);<br>Nob1(3);<br><br>H20(5);<br>H22(3);<br>H24(1);<br>H26(2);	16 of 16<br>929-944;	rpS1(5);<br>rpS13(2);<br>rpS14(1);<br><br>H20(5);<br>H22(3);<br>H24(1);<br>H26(3);
H24	51 of 51<br>977-1027;	rpS13(1);<br>rpS14(3);<br>Pno1(5);<br><br>H20(2);<br>H23(10);<br>H23a(1);<br>H25(1);<br>H27(6);<br>H45(14);	51 of 51<br>977-1027;	rpS13(1);<br>rpS14(3);<br>Pno1(5);<br>Tsr1(1);<br><br>H20(2);<br>H23(9);<br>H23a(1);<br>H25(1);<br>H27(5);<br>H45(13);

H25	22 of 22<br>1028-1037;<br>1094-1105;	rpS2(3);<br>rpS13(2);<br>rpS22(11);<br>rpS23(7);<br><br>H19(7);<br>H20(5);<br>H24(1);<br>H26(1);<br>H26a(4);<br>H45(1);	22 of 22<br>1028-1037;<br>1094-1105;	rpS13(2);<br>rpS22(11);<br>rpS23(7);<br><br>H19(7);<br>H20(6);<br>H24(1);<br>H26(1);<br>H26a(3);<br>H45(1);
H26	29 of 29<br>1038-1051;<br>1067-1081;	rpS0(1);<br>rpS1(5);<br>rpS13(1);<br>rpS22(1);<br>rpS27(4);<br>Nob1(12);<br><br>H22(5);<br>H23a(2);<br>H25(1);<br>H26_ES7(1);<br>H26a(3);	29 of 29<br>1038-1051;<br>1067-1081;	rpS1(4);<br>rpS22(1);<br>rpS27(4);<br><br>H22(6);<br>H23a(2);<br>H25(1);<br>H26_ES7(1);<br>H26a(4);
H26_ES7	13 of 15<br>1052-1058;<br>1061-1066;	rpS1(8);<br><br>H26(2);	13 of 15<br>1052-1058;<br>1061-1066;	rpS1(6);<br><br>H26(2);
H26a	12 of 12<br>1082-1093;	rpS2(1);<br>rpS21(1);<br>Nob1(2);<br><br>H2(4);<br>H19(3);<br>H25(2);<br>H26(4);<br>H36(1);	12 of 12<br>1082-1093;	 H2(4);<br>H19(3);<br>H25(2);<br>H26(4);
H27	28 of 28<br>1109-1136;	rpS23(3);<br>Tsr1(3);<br><br>H1(2);<br>H2(2);<br>H11(1);<br>H24(6);<br>H45(1);	28 of 28<br>1109-1136;	rpS23(3);<br>Tsr1(6);<br><br>H1(2);<br>H2(2);<br>H11(1);<br>H24(6);<br>H45(1);
H28	30 of 38<br>1145-1148;<br>1152-1162;<br>1616-1626;<br>1629-1632;	rpS28(3);<br><br>H2(1);<br>H29(3);<br>H35(1);<br>H36(2);<br>H43(1);	29 of 38<br>1145-1147;<br>1152-1162;<br>1616-1626;<br>1629-1632;	rpS28(4);<br>Pno1(4);<br><br>H2(2);<br>H29(2);<br>H43(1);<br>H44(1);
H29	14 of 14<br>1163-1169;<br>1575-1581;	rpS5(1);<br>Rio2(3);<br><br>H28(2);<br>H43(4);	14 of 14<br>1163-1169;<br>1575-1581;	rpS5(2);<br>Rio2(4);<br><br>H28(1);<br>H43(4);

H30	23 of 23<br>1170-1179;<br>1458-1470;	rpS15(2);<br>rpS19(1);<br>Rio2(2);<br><br>H31_unk1(2);<br>H31(1);<br>H32(1);<br>H42(8);<br>H43(2);	23 of 23<br>1170-1179;<br>1458-1470;	rpS15(2);<br>rpS19(1);<br>Rio2(4);<br><br>H31_unk1(2);<br>H42(8);<br>H43(2);
H31_unk1	6 of 6<br>1180-1185;	rpS15(3);<br>Tsr1(3);<br><br>H30(2);<br>H32(5);	6 of 6<br>1180-1185;	rpS15(5);<br>Tsr1(4);<br><br>H30(2);<br>H32(5);
H31	4 of 16<br>1186-1188;<br>1201-1201;	Tsr1(3);<br><br>H30(1);<br>H31_unk2(1);<br>H32(1);<br>H43(3);	3 of 16<br>1186-1187;<br>1201-1201;	 H31_unk2(1);<br>H32(2);<br>H43(3);
H31_unk2	7 of 7<br>1202-1208;	H31(1);<br>H32(3);<br>H42(4);<br>H43(1);	7 of 7<br>1202-1208;	H31(1);<br>H32(2);<br>H42(4);<br>H43(1);
H32	20 of 22<br>1209-1216;<br>1446-1457;	rpS15(5);<br>Enp1(2);<br>Tsr1(2);<br><br>H30(1);<br>H31_unk1(3);<br>H31(1);<br>H31_unk2(4);<br>H33(3);<br>H34(2);	17 of 22<br>1209-1216;<br>1448-1456;	rpS15(4);<br>Enp1(1);<br>Tsr1(2);<br><br>H31_unk1(3);<br>H31(1);<br>H31_unk2(3);<br>H33(2);
H33	46 of 47<br>1219-1227;<br>1229-1265;	rpS15(4);<br><br>H32(6);<br>H34(4);	42 of 47<br>1219-1227;<br>1229-1245;<br>1248-1256;<br>1258-1263;<br>1265-1265;	rpS15(4);<br>Enp1(1);<br><br>H32(3);
H34	20 of 46<br>1266-1271;<br>1278-1281;<br>1428-1430;<br>1439-1445;	Enp1(2);<br>Tsr1(1);<br><br>H32(2);<br>H33(3);	0 of 46	-
H35	15 of 15<br>1288-1293;<br>1321-1329;	rpS0(4);<br>rpS2(1);<br>Nob1(1);<br><br>H28(1);<br>H36(4);<br>H37(1);	0 of 15	-

H36	12 of 12<br>1294-1305;	rpS0(3);<br>rpS2(2);<br>Nob1(2);<br><br>H2(4);<br>H26a(2);<br>H28(1);<br>H35(4);	0 of 12	-
H37	15 of 15<br>1306-1320;	rpS0(1);<br>Nob1(4);<br><br>H35(2);	0 of 15	-
H38	10 of 15<br>1332-1337;<br>1416-1419;	rpS16(1);<br><br>H40(2);	0 of 15	-
H39	29 of 33<br>1338-1339;<br>1341-1345;<br>1347-1353;<br>1372-1386;	rpS16(2);<br><br>H39_ES9(1);<br>H40(1);<br>H41(2);	11 of 33<br>1347-1353;<br>1374-1377;	rpS16(1);<br><br>H41(2);
H39_ES9	16 of 16<br>1354-1369;	rpS16(3);<br>rpS19(8);<br><br>H39(1);	14 of 16<br>1354-1361;<br>1364-1369;	rpS16(3);<br>rpS19(7);
H40	10 of 28<br>1387-1391;<br>1408-1412;	H38(1);<br>H39(1);	0 of 28	-
H41	66 of 66<br>1471-1536;	rpS5(12);<br>rpS16(6);<br>rpS18(3);<br>rpS19(26);<br>rpS25(7);<br><br>H39(2);<br>H42(7);<br>H43(8);	66 of 66<br>1471-1536;	rpS5(12);<br>rpS16(6);<br>rpS18(3);<br>rpS19(29);<br>rpS25(7);<br><br>H39(1);<br>H42(5);<br>H43(9);
H42	38 of 38<br>1537-1574;	rpS5(1);<br>rpS15(14);<br>rpS18(14);<br>rpS19(3);<br>Rio2(4);<br><br>H30(6);<br>H31_unk2(2);<br>H41(5);<br>H43(3);	38 of 38<br>1537-1574;	rpS5(1);<br>rpS15(15);<br>rpS18(16);<br>rpS19(2);<br>Rio2(4);<br><br>H30(6);<br>H31_unk2(2);<br>H41(5);<br>H43(3);

H43	34 of 34<br>1582-1615;	rpS5(11);<br>rpS16(9);<br>rpS19(5);<br>rpS28(2);<br><br>H28(1);<br>H29(3);<br>H30(4);<br>H31(1);<br>H31_unk2(1);<br>H41(9);<br>H42(4);	34 of 34<br>1582-1615;	rpS5(12);<br>rpS16(5);<br>rpS19(5);<br>rpS28(2);<br><br>H28(1);<br>H29(3);<br>H30(4);<br>H31(1);<br>H31_unk2(1);<br>H41(8);<br>H42(4);
H44	95 of 134<br>1646-1692;<br>1710-1754;<br>1767-1769;	rpS6(5);<br>rpS8(5);<br>rpS23(2);<br>Tsr1(7);<br><br>H8(1);<br>H13(7);<br>H45(1);	94 of 134<br>1646-1692;<br>1710-1753;<br>1767-1769;	rpS6(4);<br>rpS8(7);<br>rpS23(2);<br>Tsr1(4);<br><br>H13(7);<br>H28(1);
H45	31 of 31<br>1770-1800;	rpS14(4);<br>Pno1(17);<br>Nob1(1);<br><br>H24(11);<br>H25(2);<br>H27(1);<br>H44(1);	30 of 31<br>1770-1799;	rpS14(4);<br>Pno1(17);<br>Tsr1(8);<br><br>H24(11);<br>H25(2);<br>H27(1);
