# Supplementary material for: Impact of the yeast S0/uS2-cluster ribosomal protein rpS21/eS21 on rRNA folding and the architecture of small ribosomal subunit precursors
Source: PLoS One. 2023 Mar 30;18(3):e0283698. doi: 10.1371/journal.pone.0283698 (PMC10062582; doi:10.1371/journal.pone.0283698)
Supplement: S1 Raw images — (PDF) [file pone.0283698.s008.pdf]

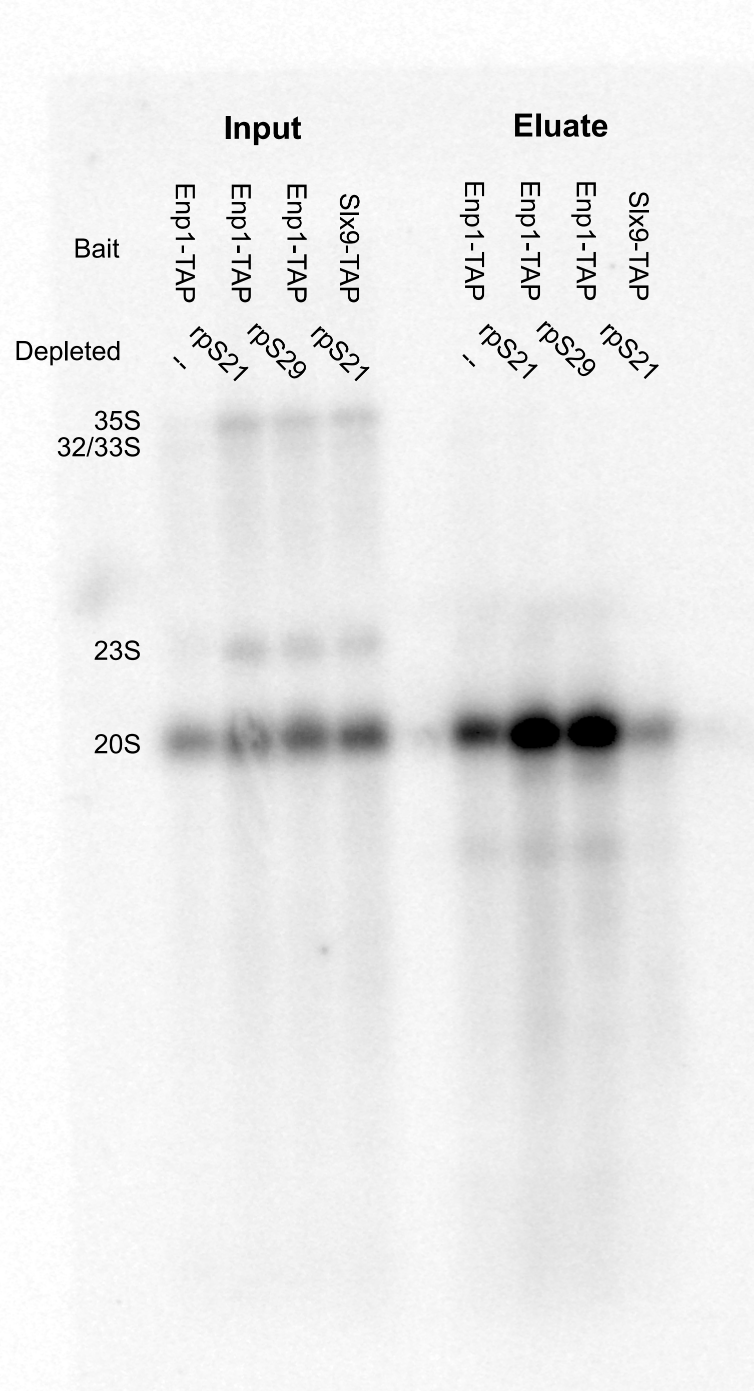


Blot exposition for S1 Figure A, probe O1819,

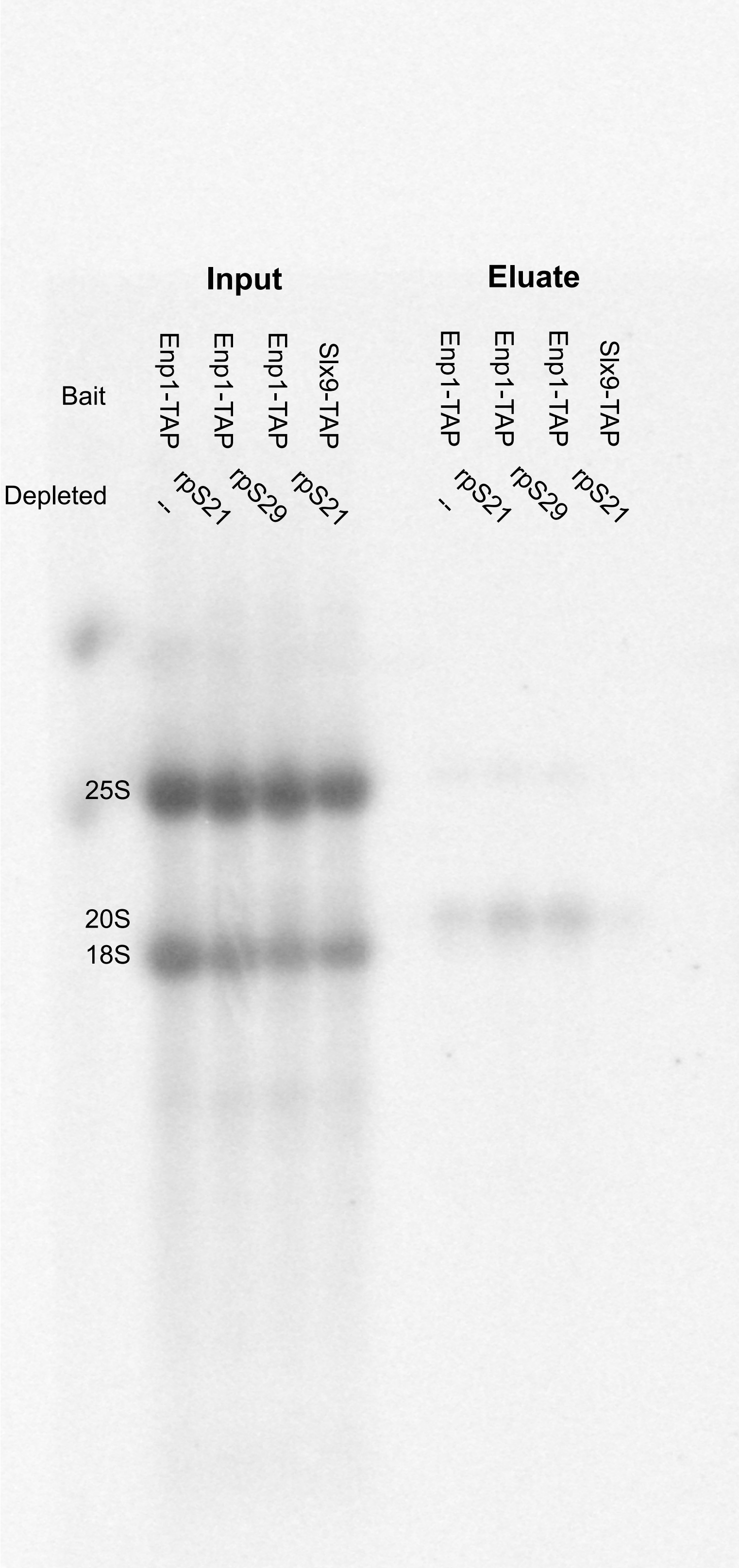


Blot exposition for S1 Figure B, probes O205+O212,

Images were generated by reading out Imaging plates exposed to 32P-labeled Northern blots with a Typhoon Imager FLA9500 (GE Healthcare)
